# Early loss to follow up after enrolment in pre-ART care at a large public clinic in Johannesburg, South Africa

**DOI:** 10.1111/j.1365-3156.2010.02511.x

**Published:** 2010-06

**Authors:** Bruce A Larson, Alana Brennan, Lynne McNamara, Lawrence Long, Sydney Rosen, Ian Sanne, Matthew P Fox

**Affiliations:** 1 Center for Global Health and Development, Boston University School of Public HealthBoston MA, USA; 2 University of the Witwatersrand, Clinical HIV Research UnitJohannesburg, South Africa; 3 University of the Witwatersrand, Health Economics and Epidemiology Research OfficeJohannesburg, South Africa

**Keywords:** HIV care, pre-antiretroviral therapy loss to follow up, South Africa

## Abstract

**Objective:**

To estimate loss to follow up (LTFU) between initial enrolment and the first scheduled return medical visit of a pre-antiretroviral therapy (ART) care program for patients not eligible for ART.

**Methods:**

The study was conducted at a public-sector HIV clinic in Johannesburg. We reviewed records of all patients newly enrolled in the pre-ART care program and not yet eligible for ART between January 2007 and February 2008. Crude proportions of patients completing their first return medical visit stratified by patient characteristics were calculated. A modified-Poisson approach was used to estimate directly relative risks of returning for their first return medical visit within 1 year adjusting for patient characteristics as potential confounders.

**Results:**

A total of 356 patients were identified. Two-thirds had a CD4 count > 350 cells/μl (median [IQR] CD4 = 458 [394, 585]) and were scheduled to return in 6 months for a first medical visit. Seventy-four percent of these patients did not return within one year for this visit. The remaining 36% of all patients had a baseline CD4 count 251–350 cells/μl and were scheduled to return in 3 months. Only 6% of these patients returned within 4 months; 41% returned within one year. Relative risks were positively associated with a patient being employed and negatively associated with the baseline CD4 count.

**Conclusions:**

Given the high rate of LTFU immediately after enroling in pre-ART care, it is clear that care programs are not expediting the timely initiation of ART. Significantly improved adherence to pre-ART care and monitoring for patients not yet eligible for ART is required for South Africa to achieve its AIDS strategy goals and reduce the problem of late presentation and initiation of ART.

## Introduction

Most HIV-infected individuals in developing countries present for HIV/AIDS care and treatment late in disease progression, with very low CD4 counts (Fairall *et al.* 2008; Rosen *et al.* 2008; Kigozi *et al*. 2009; Egger 2007). ‘Late presentation’ is defined as already eligible for antiretroviral therapy (ART) at first presentation to a treatment site, and, therefore, initiation of ART occurs well after reaching eligibility. In most developing countries, the threshold for initiating ART is already low – typically 200 CD4 cells/μl – so presentation at an even lower CD4 count poses an immediate threat to the patient's survival (Egger 2007). Given the typical time between eligibility based on CD4 counts and actual initiation of treatment, patients die or are lost to follow up before they begin therapy (Lawn *et al.* 2005, 2006; Amuron *et al.* 2009; Bassett *et al.* 2009).

Turning late presenters into ‘early presenters’– patients who enrol in pre-ART care and monitoring programs before becoming eligible for ART – requires a sequence of linked services and high retention of patients at each transition phase. This sequence starts with HIV testing, continues to CD4 testing and referral to a care program for those not ART eligible, proceeds to the patient making regular clinic visits for monitoring, and ends at ART initiation. Loss to follow up (LTFU) at any of these steps results in late presentation for treatment. We have estimated the rate of LTFU between initial enrolment in pre-ART care for patients not yet eligible for ART (baseline CD4 > 250 cells/μl) and their first scheduled return visit at one of South Africa's largest HIV/AIDS treatment facilities. This analysis complements related studies that address lost to follow up for patients who are eligible for ART but do not initiate ART (Lawn *et al.* 2005, 2006; Amuron *et al.* 2009; Bassett *et al.* 2009).

## Methods

### Study site and population

Themba Lethu Clinic (TLC) is the HIV/AIDS Comprehensive Care Management and Treatment (CCMT) facility at Helen Joseph Hospital (HJH) in Johannesburg. As of May 2009, >13 000 patients were actively on ART at TLC. The clinic is funded by the Gauteng Department of Health, with additional support from the President's Emergency Plan for AIDS Relief (PEPFAR) and the United States Agency for International Development (USAID) through Right to Care, a South African non-governmental organization (NGO).

At the time of initiating ART at TLC, patients had a median CD4 count of 98 cells/μl during 2007–2008, with 25% having a CD4 count <34 cells/μl (personal communication: M. Maskew). These figures are consistent with findings reported for other sites in South Africa and sub-Saharan Africa (Egger 2007).

In South Africa's public sector, ART eligibility is defined as a CD4 count ≤200 cells/μl or a WHO Stage IV condition. At the site, patients with CD4 counts ≤200 cells/μl are referred to the ART treatment program as well as patients with CD4 counts between 201 and 250. Those with higher counts (CD4 > 250) are not referred for treatment but instead are referred to the pre-ART care and monitoring program. Patients who enrol in pre-ART care have already made at least two visits to the clinic or elsewhere for HIV-related services–one for HIV testing and CD4 count and a second for receiving CD4 count results.

### Data collection

At TLC, enrolment in pre-ART care occurs for patients with a baseline CD4 count >250 cells/μl upon returning to the clinic for a day of wellness counselling that provides information on a range of topics including living with HIV, information on available social services, and nutrition. The clinic records a new patient's first visit to the wellness program in a logbook. The next step in pre-ART care is to return to the clinic for a first follow-up medical visit with a primary health care nurse. Patients are scheduled to return for this visit at 3 months if their enrolment CD4 count is 250–350 cells/μl or at 6 months if their enrolment CD4 count is >350 cells/μl.

Wellness logbooks were reviewed to identify all new patients enroling in pre-ART care at TLC from January 2007 to February 2008. Patient records were reviewed in June 2009 to determine whether each subject returned for their first medical visit. Visit dates and demographic information from patient records were also recorded. Censoring of the dataset in June 2009 provided a minimum follow-up of 15 months for all subjects. Patients with a baseline CD4 count ≤ 250 cells/μl were not included in this analysis because they are managed differently in preparation for ART initiation.

The study received ethical approval from Boston University and the University of the Witwatersrand.

### Statistical analysis

Patients with CD4 counts of 250–350 cells/μl were scheduled to return at 12 weeks. For these patients, we defined two primary outcomes: whether or not the subject returned to TLC (1) within 4 and 40 weeks of their scheduled return appointment (16 week and 52 week total return period after enrolment). We also defined two primary outcomes for patients with CD4 counts >350 who were scheduled to return at 24 weeks: whether or not the subject returned to TLC within 12 and 28 weeks of their scheduled return appointment (36 and 52 week total return period after enrolment). Crude proportions of patients completing their first return medical visit based on these primary outcomes were calculated.

We estimated directly relative risks of returning for CD4 results within 1 year using the modified Poisson approach developed by Zou (2004). A log-binomial approach can also be used for estimating relative risks directly, although the approach may estimate standard errors that are too small and convergence problems may occur (Mcnutt *et al.* 2003). A simple Poisson approach, on the other hand, is known to provide estimates of standard errors that are too large (Zou 2004). The modified Poisson approach simply applies a standard Poisson model with robust standard errors. The relative risk model includes the following patient-specific factors as potential confounders (age, sex, marital and employment status, and baseline CD4 count).

## Results

A total of 544 patients were newly enrolled in the TLC pre-ART care program during the study period. We excluded 41 patients who enrolled in the program but did not have an enrolment CD4 count and 147 patients indicated in the log book as not yet eligible for ART, but who were later found to have a CD4 count ≤250 cells/μl at enrolment as these patients were not early presenters. This left 356 patients for analysis.

**Figure 1:**
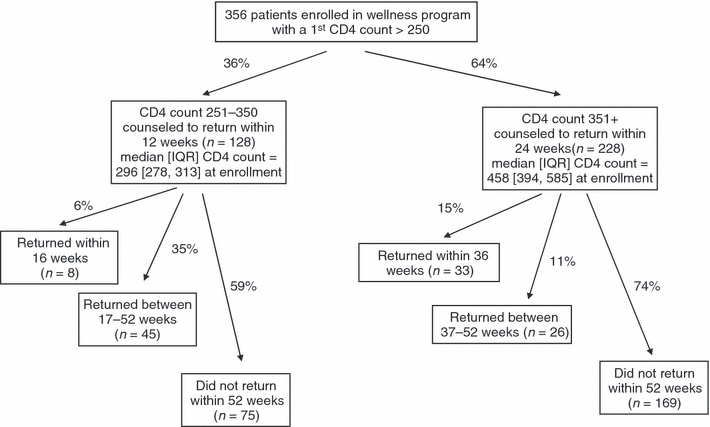
Lost to follow up among patients newly enrolled in pre-ART HIV care (did not return for first care program medical visit).

Figure 1 summarizes the crude proportions stratified by baseline CD4 count of patients (expressed as percentages) that were loss to follow up. Of the 356 total patients, 128 (36%) presented with a CD4 count ≤350 cells/μl (median [IQR] = 296 [278–313]). These patients were counselled to return within 3 months for their first medical visit. Eight (crude proportion 6%; 95% CI 2.3%–11.5%) completed the visit within 4 months of staging, 45 (crude proportion 35.2%; 95% CI 27.3%–43.7%) returned within 1 year, and the remaining 75 (crude proportion 59%; 95% CI 50.0%–67.0%) did not return within a year.

The other 228 patients (64%) enrolled with a CD4 count >350 cells/μl (median [IQR] = 458 [394–585]) and were counselled to return within 6 months for their initial medical visit. Of these, 33 (crude proportion 14.5%; 95% CI 10.3%–19.5%) completed the visit within 9 months, 26 (crude proportion 11.4%; 95% CI 7.7%–16.0%) within a year and the remaining 169 (crude proportion 74%; 95% CI 68.1%–79.5%) did not return within a year (Figure 1).

Table 1 provides basic summary information on key patient characteristics stratified by those who returned within one year and those who did not. In both groups, median age was 35 years, 21% were married, and about 25% were men. A higher proportion of patients who returned within one year were employed (57% compared to 44%). The median CD4 count was lower for patients who returned within one year than for those who did not (354 compared to 403).

**Table 1:** Descriptive statistics for those who returned for the first care program medical visit within 52 weeks and those who did not (% unless otherwise specified)

Variable	Returned within 52 weeks	Did not return within 52 weeks	*P*-value*
Age (median [IQR])	35 [30, 42]	35 [30, 42]	0.68
Employed	57%	44%	0.02
Male	24%	27%	0.55
Married	21%	21%	0.94
CD4 (median [IQR]	354 [301, 455]	403 [321, 550]	0.01

* *P*-value for *t*-test of equality of sample means (in this case proportions) assuming unequal variances or non-parametric two-sample test on the equality of medians (using *t*-test and median command in Stata 10.1).

Relative risks based on the modified Poisson model are reported in Table 2. Baseline CD4 count and employment status were significant predictors of returning for the first medical visit. Compared to the group with the lowest baseline CD4 count (251–350 cells/μl), the risks of returning within 1 year dropped substantially (RR: 0.66; 95% CI: 0.46–0.96) when the baseline CD4 count was 351–450 cells/μl and 0.61 (95% CI 0.42–0.88) for CD4 > 450 cells/μl. The risk of returning was greater for employed patients (RR 1.41; 95% CI 1.03–1.92) *vs.* unemployed patients.

**Table 2:** Relative risks of returning for the first medical visit*

Returned 52 weeks	Relative Risk	*P*-value	95% Confidence Interval	
Age	1.005	0.517	0.990	1.020
Employed	1.408	0.031	1.032	1.922
Male	0.834	0.332	0.578	1.203
Married	0.939	0.746	0.643	1.371
cd4 251–350	†	†	†	†
cd4 351–450	0.661	0.028	0.457	0.956
cd4 451+	0.606	0.009	0.417	0.883

* Relative risk estimated based on Poisson regression with robust standard errors in STATA 10.1 following Zho (Zou 2004), adjusted for age, employed, male, married, and CD4 categories specified in the previous table.

† The reference case.

## Discussion

In this study, 69% of patients enrolled in pre-ART care failed to return for their first medical visit within 1 year of enrolment. This highlights the need for greater investment in patient retention from the beginning of HIV care, not just after ART begins.

Our data, combined with a previous analysis of post-voluntary counseling and testing (VCT) rates of CD4 testing at the same site (Larson *et al.* 2009), paint a striking picture. First, very few patients (13%) who learn their HIV status prior to ART eligibility return to the clinic to obtain their CD4 counts (Larson *et al.* 2009). Second, we showed only a small proportion of pre-ART care patients return for their first medical visit on time; less than 1/3 of these patients return within 1 year of enrolment.

According to the *HIV/AIDS and STI Strategic Plan for South Africa, 2007–2011* (South African National Department of Health), South Africa aims to ‘provide an appropriate package of treatment, care and support services to 80% of HIV positive people and their families by 2011 in order to reduce morbidity and mortality as well as other impacts of HIV/AIDS’ (South African National Department of Health, p. 57). One of the main goals of pre-ART care is to monitor disease progression to ensure as early a start on ART as possible, hopefully before the patient develops serious clinical illness. The low rate of retention we observed at the very beginning of the HIV care program does not bode well for achieving the country's stated goal. Patients who do not even return for their first medical visit may have benefited by being made aware of their status and from the counselling that accompanied the HIV test and CD4 count, but they are still at risk of becoming late presenters. Pre-ART care thus remains a broken link in the chain that should connect HIV testing to ART through pre-ART care. This link must be repaired if the problem of late presentation for treatment is to be solved and South Africa is to meet its national policy goals.

This study had several limitations. It was conducted at only one facility in South Africa, albeit one of the largest, so it is possible that experience differs at other sites. Importantly, there is no medical information system in South Africa that allows patients to be tracked from one facility to another. We cannot therefore rule out the possibility that some of our subjects transferred to a care program elsewhere, though this is unlikely to explain more than a fraction of the high level of attrition we observed. Finally, we do not know why the subjects in our study did or did not return. It is possible that some patients with low CD4 counts who should have returned within 3 months but instead waited 9–10 months, returned because they were sick and needed medical care. Patients like these, who can no longer accurately be labelled ‘early presenters,’ may cause our results to overestimate the proportion of patients who adhered to the program schedule.

None of these limitations is sufficient to alter the core finding of this study, which is that the majority of patients are lost from pre-ART care between enrolment and their first return visit. Future research is needed to examine why patients are LTF in pre-ART care and what interventions are feasible and effective for reducing such losses. We suspect that barriers to returning for pre-ART program visits are similar to barriers to ART adherence in general, which include, but are not limited to, costs and/or time for transport, lost earnings, lack of financial resources, stigma, lack of family support, perceived need and psychological issues (Jones 2005; Mshana *et al.* 2006; Horne *et al.* 2007; Kinsler *et al.* 2007; Makombe *et al.* 2007; Muula *et al.* 2007; Wilson & Blower 2007). Employed patients were substantially more likely to complete their return medical visit within one year than unemployed patients, which is consistent with the literature on barriers to ART adherence in general. Future research should focus on identifying these barriers for particular patient populations and seeking strategies for overcoming them.
